# Differential contribution of genomic regions to marked genetic variation and prediction of quantitative traits in broiler chickens

**DOI:** 10.1186/s12711-016-0187-z

**Published:** 2016-02-03

**Authors:** Rostam Abdollahi-Arpanahi, Gota Morota, Bruno D. Valente, Andreas Kranis, Guilherme J. M. Rosa, Daniel Gianola

**Affiliations:** Department of Animal Sciences, University of Wisconsin, Madison, WI USA; Department of Animal and Poultry Science, College of Aburaihan, University of Tehran, Pakdasht, Iran; Department of Animal Science, University of Nebraska, Lincoln, NE USA; Department of Dairy Science, University of Wisconsin, Madison, WI USA; Aviagen Ltd, Midlothian, UK; The Roslin Institute and Royal (Dick) School of Veterinary Studies, University of Edinburgh, Midlothian, UK; Department of Biostatistics and Medical Informatics, University of Wisconsin, Madison, WI USA

## Abstract

**Background:**

Genome-wide association studies in humans have found enrichment of trait-associated single nucleotide polymorphisms (SNPs) in coding regions of the genome and depletion of these in intergenic regions. However, a recent release of the ENCyclopedia of DNA elements showed that ~80 % of the human genome has a biochemical function. Similar studies on the chicken genome are lacking, thus assessing the relative contribution of its genic and non-genic regions to variation is relevant for biological studies and genetic improvement of chicken populations.

**Methods:**

A dataset including 1351 birds that were genotyped with the 600K Affymetrix platform was used. We partitioned SNPs according to genome annotation data into six classes to characterize the relative contribution of genic and non-genic regions to genetic variation as well as their predictive power using all available quality-filtered SNPs. Target traits were body weight, ultrasound measurement of breast muscle and hen house egg production in broiler chickens. Six genomic regions were considered: intergenic regions, introns, missense, synonymous, 5′ and 3′ untranslated regions, and regions that are located 5 kb upstream and downstream of coding genes. Genomic relationship matrices were constructed for each genomic region and fitted in the models, separately or simultaneously. Kernel-based ridge regression was used to estimate variance components and assess predictive ability. Contribution of each class of genomic regions to dominance variance was also considered.

**Results:**

Variance component estimates indicated that all genomic regions contributed to marked additive genetic variation and that the class of synonymous regions tended to have the greatest contribution. The marked dominance genetic variation explained by each class of genomic regions was similar and negligible (~0.05). In terms of prediction mean-square error, the whole-genome approach showed the best predictive ability.

**Conclusions:**

All genic and non-genic regions contributed to phenotypic variation for the three traits studied. Overall, the contribution of additive genetic variance to the total genetic variance was much greater than that of dominance variance. Our results show that all genomic regions are important for the prediction of the targeted traits, and the whole-genome approach was reaffirmed as the best tool for genome-enabled prediction of quantitative traits.

## Background

To date, analysis of pathways and post-genome-wide association studies (GWAS) have focused on genic regions of the genome as evidenced by the emergence of exome sequencing. Exons are functional sequences of the genome which, taken together, represent an important part of the genome that is actually translated into protein. Moreover, genotyping exons is less expensive than whole-genome sequencing. However, a recent release of the ENCyclopedia of DNA Elements (ENCODE) showed that about 62 % of the genome is transcribed into RNA, which added to the evidence that has accumulated on transcription-factor-binding sites, chromatin structure, DNA methylation, histone modification and other regulatory regions, indicates that about 80 % of a genome has a biochemical function [1]. Nonetheless, DNA sequences in intergenic regions are considered as “dark matter” or “dark matter transcripts” [2] since their role is still ambiguous. Recent research has shown that 43 % of the regions that are detected in GWAS point to intergenic regions (outside of the promoter and transcribed regions), and 45 % to introns [3]. Nevertheless, missense codons and promoter regions are significantly enriched for trait-associated single nucleotide polymorphisms (SNPs), while intergenic regions are significantly underrepresented [3, 4].

On the one hand, most GWAS have used very stringent significance thresholds to avoid false positives due to multiple-testing and, as a result, many variants with small effects have been missed. These also include rare variants that have large effects but explain a small proportion of the variance [5]. On the other hand, in whole-genome prediction, the prediction of genetic merit of individuals is based on the effect of all variants estimated simultaneously. Such an approach does not suffer from multiple-testing, stringent significance thresholds and unrealistic assumptions like linkage equilibrium (LE) between markers, since linkage disequilibrium (LD) is pervasive, especially for agricultural species.

The contribution of genic and non-genic regions of the genome to additive genetic variance has been investigated in humans [6–8], dairy and beef cattle [9] and plants [10]. There is, however, some disagreement between the findings from these studies. For instance, Yang et al. [8] stated that genic regions contributed more additive genetic variation than non-genic regions. Koufariotis et al. [9] also pointed out that the classes of missense and synonymous genomic regions explained most of the additive genetic variation. On the contrary, Gusev et al. [7] reported that DNaseI hypersensitivity sites explained most of the additive genetic variation for 11 common diseases. However, a study by Do et al. [11] on feed intake and its component traits in pigs indicated that the contribution of each SNP to total genomic variance was similar for genic and non-genic regions.

Morota et al. [12] studied the predictive ability of various genomic regions for three chicken broiler traits. They found that the enrichment or depletion of genomic regions in terms of predictive ability was trait-dependent and that the whole-genome approach had the best predictive power regardless of trait. Erbe et al. [13] compared the predictive ability of SNPs in transcribed regions with that of SNPs in intergenic regions and found that the transcribed part of the genome of dairy cattle performed better, with a 0.03 increase in predictive correlation for Jersey cattle traits. However, these studies fitted genic and non-genic regions using a single kernel (single genomic relationships matrix) approach. An alternative approach would be to use multiple kernels (multiple genomic relationship matrices) that are tailored to each specific genomic region.

Partitioning genetic variation into marked additive and dominance components has been explored [14–18] but knowledge on the contribution of different genomic regions to non-additive genetic variation is lacking. Partitioning the genome into classes of SNPs allows one to target genomic regions of interest.

While Morota et al. [12] used the same dataset to evaluate the predictive performance of different genomic regions, some limitations of their study led us to conduct additional research, i.e.: (1) they used a single-kernel approach, while the multiple-kernel approach is more flexible for prediction and, thus, can be used to improve accuracy of prediction and to decrease learning complexity and training time e.g., [19]; (2) the non-additive contribution of different genomic regions was not considered; (3) variance components estimates were not studied; and (4) overlapping genic regions complicated the interpretation of results, thus partitioning the genome into distinct segments may produce a clearer picture. Therefore, the aim of our study was to investigate the relative contribution of genic and non-genic regions to marked additive and dominance genetic variation for body weight (BW), breast muscle (BM) and hen house egg production (HHP) in broiler chickens. We also evaluated the predictive ability of different genomic regions for yet-to-be observed phenotypes. To our knowledge, this is the first study that addresses the contribution of genomic regions to marked non-additive genetic variation by fitting one class of genomic regions at a time.

## Methods

### Data

A total of 1351 birds from a commercial broiler chicken line were provided by Aviagen Ltd. This broiler line has undergone several generations of selection using genetic evaluations based on multiple-trait pedigree best linear unbiased prediction (P-BLUP). The following traits were studied: BW, ultrasound of BM at 35 days of age, and HHP defined as the total number of eggs laid between weeks 28 and 54 per bird. Phenotype records for BW and BM were pre-corrected for a combined effect of sex (525 males and 826 females), hatch week, contemporary group of parents and pen in the growing farm, whereas phenotype records for HHP were adjusted for hatch effects. Phenotypic records were merged with SNP genotype records on individuals that were genotyped using the Affymetrix 600K chip. More details on genotyping and phenotypic data are in Kranis et al. [20] and Abdollahi-Arpanahi et al. [21]. In total, there were 1346, 1331, and 819 individuals scored for BW, BM and HHP, respectively.

A total of 580,954 SNP genotypes were originally available in the dataset. SNPs that departed from Hardy–Weinberg equilibrium (p < 10^−6^) based on a Chi square test, SNPs that had a minor allele frequency (MAF) <0.01, or a missing rate >0.05 were excluded from the analysis. Missing SNPs were imputed using Beagle [22]. After editing, 354,364 SNPs remained for the analysis. Mean MAF was equal to 0.27. Only SNPs on 28 autosomes (GGA1–28, GGA for *Gallus gallus* chromosome) were included, which covered 919 Mb of the *G. gallus* genome. Data editing was done with the PLINK software [23]. The following coding was used for SNP substitution effects in the additive genotype matrix (**X**_A_) i.e. 0 for “aa”, 1 for “Aa”, and 2 for “AA”.

### SNP annotation

Chromosome information and physical positions of SNPs were obtained using the annotation file downloaded from the Animal Genome Database (http://www.animalgenome.org/repository/chicken/). We mapped the Aviagen marker coordinates file to Gallus_gallus_4.0 Ensembl VEP tool (release 75) (ftp://ftp.ensembl.org/pub/release-75/variation/VEP/arrays/) and to the animal genome annotated file of chicken data. Each SNP was examined to determine if it was located in genic or non-genic regions. Six classes of disjoint genomic regions were formed, namely, introns, missense (non-synonymous), synonymous, 5′ and 3′ untranslated regions (UTR), regions that are located 5 kb upstream and downstream of gene (“up-down” class), and intergenic regions, which included SNPs that were not annotated as being in genic regions. Missense genomic regions are regions where a point mutation causes a single nucleotide change in a codon that codes for a different amino acid. Conversely, the annotation class of synonymous regions includes SNPs for which the substitution of one base for another in a coding region does not modify the resulting amino acid. Regulatory regions were defined as regions located 5 kb upstream and downstream of genes. In our study, intergenic regions consisted of SNPs without any assignment to the aforementioned annotation categories. Numbers of SNPs and their allelic frequency for each class of genomic regions are in Table 1. After quality control, 43,600 SNPs were not found in the Ensembl database or animal genome annotated database and were discarded from all further analyses. Finally, 310,764 SNPs were retained for the final analysis.

**Table 1:** Number of SNPs assigned to each genomic region and the corresponding mean and standard deviations (SD) of minor allelic frequencies (MAF)

Classes of genomic regions	Number of SNPs	Mean MAF	SD MAF
Intergenic	139,394	0.27	0.13
Intron	124,734	0.27	0.13
Missense	1658	0.27	0.13
Synonymous	5620	0.27	0.13
UTR	3044	0.28	0.13
Upsteam and downstream	36,314	0.27	0.13
All markers	310,764	0.27	0.13

### Statistical models

To explore the variance and predictive ability of each of the six aforementioned classes of genomic regions, 12 additive and dominance parametric kernels were constructed. Kernel methods have been reviewed in Gianola et al. [24], Gianola and van Kaam [25], de los Campos et al. [26] and Morota and Gianola [27]. In order to capture signals from genotypes to phenotypes through the construction of kernel matrices **G** (additive genomic relationships) and **D** (dominance genomic relationships), three scenarios were considered: (1) fitting additive kernels (**G**) for each genomic region or fitting one kernel (**G** matrix) for all SNPs without distinction of genomic regions; (2) fitting additive and dominance kernels (**G** + **D**) jointly for each genomic region; and (3) fitting six additive kernels jointly $({\mathbf{G}}_{1} + {\mathbf{G}}_{2} + \cdots + {\mathbf{G}}_{6} ),$ with each kernel linked to one genomic region. All additive kernels are parametric (linear) kernels that were constructed following VanRaden’s [28] genomic relationship matrices based on SNP information. While a joint analysis that involves 12 additive and dominance kernels is appealing, it was not considered here because in terms of convergence issue, it is too computationally demanding for at least two reasons: (1) small sample size; and (2) poor mixing since the additive and dominance kernels were not orthogonal to each other. Each of the statistical models is described below.

#### Scenario (1): Separate analysis with additive kernels (**G**)

In this analysis, we fitted models by accounting for the similarity relationship matrix (kernel) within each of the six classes of genomic regions. Phenotypes that were pre-corrected for systematic effects were analyzed trait by trait with the following model:

1 $${\text{y}}_{\text{i}} =\upmu + {\text{g}}({\mathbf{x}}_{\text{i}} ) + {\text{e}}_{\text{i}} ,$$

where y_i_ is the pre-corrected phenotype on bird i; μ is the intercept; ${\text{g}}({\mathbf{x}}_{\text{i}} )$ is a linear function of SNP genotypes **x**_i_, and e_i_ is the residual of the model for bird i. We assumed that the genetic signals (***g***) were represented by **Gα**, where **G** is an n × n kernel matrix indexed by the observed SNP covariates such that **G** ~ **XX**^T^, where **X** is a SNP genotype matrix. **G** resulted from a centered and standardized **X** matrix, then divided by the number of SNPs, as proposed by VanRaden [28] and Yang et al. [29], and **α** is the vector of the reproducing kernel Hilbert spaces (RKHS) regression coefficients that is estimated as the solution that minimizes:

$$l({\varvec{\upalpha}}|\lambda ) = (\varvec{y} - {\mathbf{G}}{\varvec{\upalpha}})^{\prime } (\varvec{y} - {\mathbf{G}}{\varvec{\upalpha}}) + \lambda {\varvec{\upalpha}}^{\prime } \varvec{G}{\varvec{\upalpha}},$$

where $${\varvec{\upalpha}}\sim\,{\text{N(0,}}\,{\mathbf{G}}^{ - 1} \sigma_{\text{g}}^{2} ),$$$$\sigma_{\text{g}}^{2}$$ is the additive variance captured by markers, and *λ* is a regularization factor. Since ***g*** = **Gα**, the hundreds of thousands of SNP predictors are featured into a number of functions that is equal to the number of observations, i.e. 1346, 1331 or 819 individuals for BW, BM, and HHP, respectively.

We can now rewrite Eq. (1) in matrix form as:

$${\mathbf{y}} = {\mathbf{1}}\upmu + {\mathbf{G}}{\varvec{\upalpha}} + {\mathbf{e}},$$

where **1** is a vector of ones with the appropriate dimension, and **e** is a vector of model residuals with **e** ~ N(0, **I**$\sigma_{\text{e}}^{2}$), where $\sigma_{\text{e}}^{2}$ is the residual variance. We assume that the variance–covariance structure of the above model was:

$${\mathbf{V}} = {\mathbf{G}}\sigma_{\text{g}}^{2} + {\mathbf{I}}\sigma_{e}^{2} .$$

The prediction of genetic values in a testing set $(\hat{\varvec{g}}_{test} )$ is given by:

$$\hat{\varvec{g}}_{test} = {\mathbf{G}}_{test,strain} \varvec{G}_{train}^{ - 1} \hat{\varvec{g}}_{train} ,$$

where ${\mathbf{G}}_{test,strain}$ is a rectangular matrix of genomic relationships between training and testing individuals, which represents a subset of the total **G** constructed from all individuals in the training and testing sets, ***G***_*train*_ is the genomic relationship between individuals in the training set, and $\hat{\varvec{g}}_{train}$ is the vector of predicted genetic signals of individuals in the training set.

#### Scenario (2): Joint analysis of additive and dominance kernels (**G** + **D**) for each genomic region

The standard RKHS regression model can be represented in this case as:

2 $${\mathbf{y}} = {\mathbf{1}}\mu + {\mathbf{G}}{\varvec{\upalpha}}_{1} + {\mathbf{D}}{\varvec{\upalpha}}_{2} + {\text{e}} .$$

where ${\varvec{\upalpha}}_{1} \sim\,N(0,{\mathbf{G}}^{ - 1} \sigma_{g}^{2} )$ and ${\varvec{\upalpha}}_{2} \sim{\text{N}}(0,{\mathbf{D}}^{ - 1} \sigma_{d}^{2} )$ are unknown regression vectors in RKHS, where $\sigma_{d}^{2}$ is the marked dominance variance and **D** is the dominance relationship matrix. To build **D**, we created an incidence matrix (**X**_**D**_) for effects due to dominance **X**_**D**_ = (x_Dij_). Elements of **X**_**D**_ are equal to −2*q*^2^, 2*pq* and −2*p*^2^ for genotypes aa, Aa and AA, respectively, where *p* is the frequency of A and *q* = 1 − *p*. Then, ${\mathbf{X}}_{{\mathbf{D}}} {\mathbf{X}}_{{\mathbf{D}}}^{\prime }$ is standardized at $4\sum\nolimits_{i = 1}^{m} {p_{i}^{2} q_{i}^{2} }$ as in Vitezica et al. [30], where *m* is the number of SNPs. With this structure of **G** and **D**, the variance–covariance structure was:

$${\mathbf{V}} = {\mathbf{G}}\sigma_{\text{g}}^{2} + {\mathbf{D}}\sigma_{d}^{2} + {\mathbf{I}}\sigma_{e}^{2} .$$

The additive genetic values of individuals in the testing set were predicted with the equation in Scenario (1), and $\hat{\varvec{d}}_{test} = {\mathbf{D}}_{test,train} \varvec{D}_{train}^{ - 1} \hat{\varvec{d}}_{train}$ was used for dominance values.

#### Scenario (3): Joint analysis of six additive kernels $({\mathbf{G}}_{1} + {\mathbf{G}}_{2} + \cdots + {\mathbf{G}}_{6} )$

In the joint analysis of the six classes of genomic regions, we used the following statistical model:

3 $${\mathbf{y}} = {\mathbf{1}}\mu + \sum\limits_{t = 1}^{h} {{\mathbf{G}}_{t} {\varvec{\upalpha}}_{t} + {\mathbf{e}}} ,$$

where **y** is the vector of observations; **1** is a vector of ones; μ is an intercept; h = 6 is the number of genomic regions; **α**_t_ ~ (**0**, ${\mathbf{G}}_{t}^{ - 1} \sigma_{gt}^{2}$) with ${\text{t}} = 1,2, \ldots ,6$ is a regression vector in RKHS for genomic region t; **G**_t_ is a matrix of additive genomic relationships for the* t*th genomic region; $\sigma_{{g_{t} }}^{2}$ is the variance that is captured by SNPs in the *t*th genomic region; and ${\mathbf{e}}\sim {\text{N(}}0,{\mathbf{I}}\sigma_{e}^{2} )$ is a vector of model residuals, where $\sigma_{e}^{2}$ is the residual variance, and **I** is an identity matrix. Therefore, the joint density of the six random vectors and of the residual term is:

$$p\left( {{\mathbf{e}},{\mathbf{g}}_{1} , \ldots , {\mathbf{g}}_{t} |\sigma_{e}^{2} , \sigma_{{g_{1} }}^{2} , \ldots ,\sigma_{{g_{t} }}^{2} } \right) = N\left( {{\mathbf{e}}|0,{\mathbf{I}}\sigma_{e}^{2} } \right)\mathop \prod \limits_{t = 1}^{h} N\left( {{\mathbf{g}}_{t} |0,{\mathbf{G}}_{t} \sigma_{{g_{t} }}^{2} } \right).$$

The marginal distribution of the data in model (3) has an expected value of **1**μ and the variance–covariance matrix is ${\mathbf{V}} = \sum\nolimits_{{{\text{t}} = 1}}^{\text{h}} {{\mathbf{G}}_{\text{t}} \sigma_{\text{gt}}^{2} + {\mathbf{I}}\sigma_{\text{e}}^{2} .}$ It was assumed that there was no covariance between effects of SNPs from different genomic regions. Prediction of genetic value obtained with SNPs in region t for individuals in the testing set was as follows:

$$\hat{\varvec{g}}_{t,test} = {\mathbf{G}}_{t, test,train} \varvec{G}_{t,train}^{ - 1} \hat{\varvec{g}}_{t,train} ,$$

where notations are as in Scenario (1), except that t indicates the **G** matrix of *t*th genomic region.

To illustrate the difference or similarity in information that is captured by the additive genomic relationships in each of the classes of genomic regions, the Euclidean distance (ED) between each pair of genomic relationships was calculated using the formula:

4 $$ED = \sqrt {\sum\nolimits_{i = 1} {\left( {a_{i} - b_{i} } \right)}^{2} } ,$$

where *a* and *b* are the corresponding elements of the genomic relationship matrices for each annotation class. This distance matrix was subsequently fed to an R package for clustering and drawing heat map plots [31].

### Implementation of Bayesian analysis

To implement the procedures within a Bayesian framework, a flat prior was assigned to μ, and independent scaled inverse Chi square distributions were assigned to the variance components. The hyper-parameters for each of the inverse Chi square distributions were equal to 5 for degrees of freedom and the scale parameter was calculated according to the default value of the BGLR package [32]. All analyses were conducted using RKHS regression as implemented in the BGLR package.

The Bayesian model was run via Gibbs sampling. For each fitted model, a Markov chain Monte Carlo (MCMC) with 500,000 samples was run and the first 50,000 samples were discarded as burn-in. Subsequently, 450,000 samples were obtained and thinned at a rate of 50, resulting in 9000 mildly correlated samples for posterior inference. Convergence diagnostics and statistical and graphical analysis of Gibbs sampling were checked by visual inspection of trace plots of some parameters (i.e. variance components) and with the Coda [33] package.

### Predictive ability

The predictive ability of our RKHS regression models was assessed by cross-validation (CV). Specifically, a fourfold CV scheme was applied by assigning animals randomly to one of four separate subsets. Of these four subsets, three were combined to form a training set and one was used as testing set. Each of the four subsets was used as a testing set only once. Since the CV distribution was dispersed because of the small size of the sample, the above fourfold CV was replicated 15 times, at random, and results were averaged over replications. Predictive abilities were assessed via Pearson’s product-moment correlation between pre-adjusted phenotypes and predicted phenotypes (predicted genetic values plus intercept), and via prediction mean-squared error (MSE).

## Results

### Variance components

**Fig. 1:**
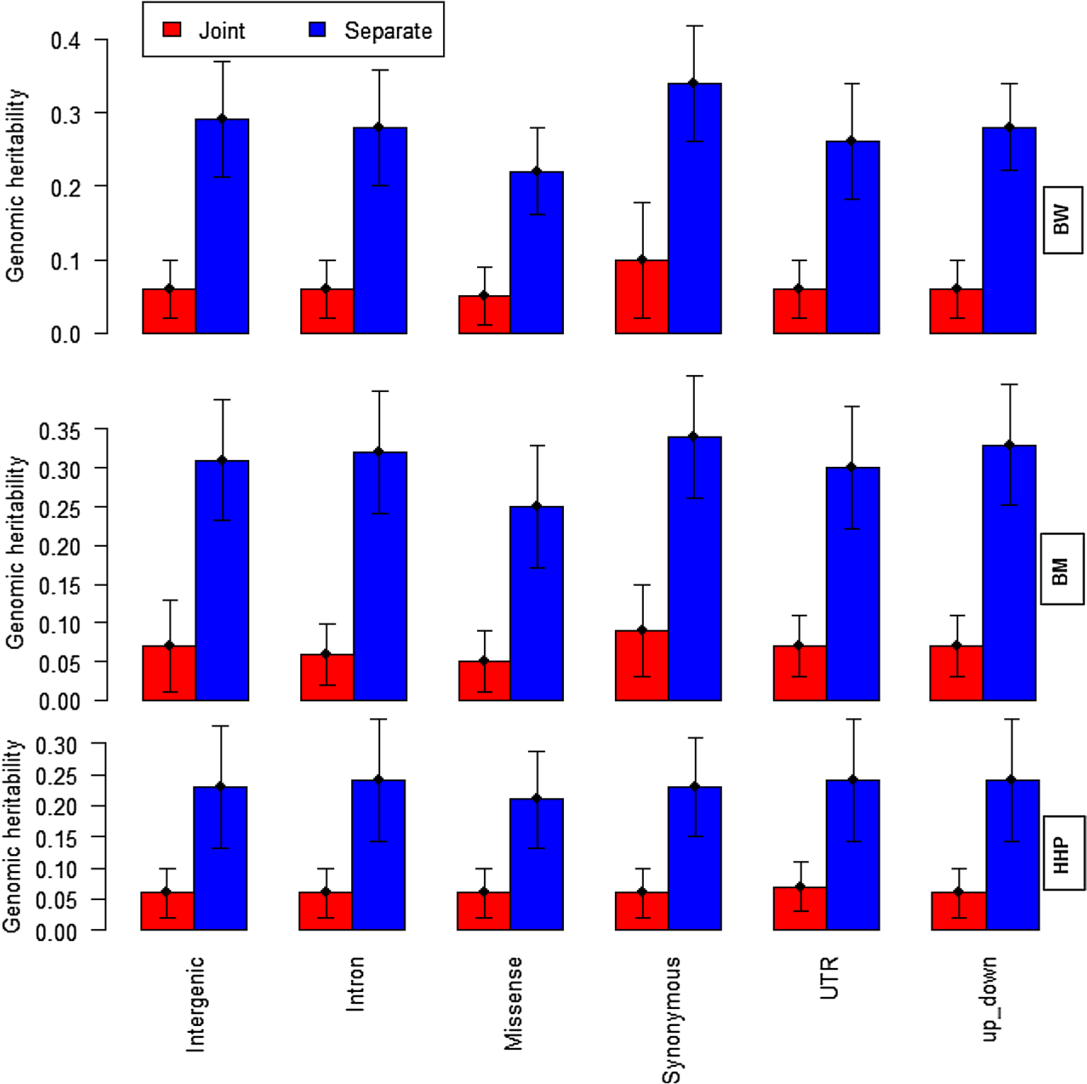
Marked genomic heritability estimates with SNPs partitioned into six classes of genomic regions for body weight (BW), ultra-sound of breast muscle (BM) and hen house egg production (HHP). *Red bars* and *blue bars* show joint and separate analyses, respectively. “*up*-*down*” indicates regions that are located 5 kb upstream and downstream of the gene. The *whiskers* represent 95 % confidence interval

Narrow-sense genomic heritabilities were estimated by fitting all SNPs together and were equal to 0.29 ± 0.04 for BW, 0.33 ± 0.04 for BM and 0.24 ± 0.04 for HHP. These estimates agreed with those from our previous study using the same dataset but a different number of SNPs and a restricted maximum likelihood approach to estimate variances [21]. The genomic heritability associated with each class of genomic regions in the separate (Scenario 1) and joint analyses (Scenario 3) is presented in Fig. 1. Estimates obtained by fitting the six classes of genomic regions separately or together differed significantly: i.e. estimates from the separate analyses were much larger than those from the joint analysis for all classes of genomic regions and all traits. In the separate analyses, the variance attributable to each class of genomic regions was overestimated because of the LD between SNPs in different regions, whereas in the joint analysis, all classes of genomic regions acted together and leveraged the polygenic basis of each trait, possibly resulting in more accurate estimates. The mean LD (r^2^) 0.32 was observed for SNPs that were separated by <20 kb and it dropped to 0.21 when the distance between SNPs reached 100 kb for the current population. This amount of r^2^ was almost the same within and between SNPs in different genomic regions.

Interestingly, in both analyses, all classes of genomic regions made significant contributions to the narrow-sense genomic heritability with the class of synonymous regions having a slightly stronger impact. If the estimates of narrow-sense genomic heritability for each class of genomic regions in the separate analysis are added up, the genomic heritability would also be out of range. However, in the joint analysis, the sum of the six component estimates was similar to the estimate from the whole-genome analysis for each trait. This reinforces the concept that LD causes single SNP regression (GWAS) to capture effects due to other SNPs, and the same is observed with variance component estimates.

In the joint analysis of BW, the estimated genomic heritability ranged from 0.05 to 0.10 for the different classes of genomic regions (Fig. 1). The largest estimate was attributed to the class of synonymous regions. For BM (joint analysis, Fig. 1), the estimates ranged from 0.05 for the class of missense regions to up to 0.09 for that of synonymous regions, and the distribution of the estimates of genomic heritability over the six classes for BM was similar to that for BW. For HHP (joint analysis, Fig. 1), the estimated genomic heritability ranged from 0.06 for the class of missense regions to 0.07 for the UTR class. Differences in the relative contribution of each class of genomic regions were similar in the joint and separate analyses. However, the difference in estimates of genomic heritability between classes of genomic regions was larger in the separate analysis than in the joint analysis. The posterior standard deviations of the genomic heritability in the separate analysis were larger than in the joint analysis.

Although the number of SNPs within coding DNA regions (synonymous and missense) was smaller than in non-coding regions (introns and intergenic), the magnitude of the genetic variance for these regions was similar and, in some cases, larger than for the non-coding regions. In order to check the influence of number of SNPs within a class of genomic regions, we picked 1500 random SNPs from each class and performed the same analysis to estimate genetic parameters with ten replications. The results (not shown here) produced a similar pattern as those for all the SNPs within each class. Hence, we concluded that our results were neither driven by differences in allelic frequency distributions, which were similar (Table 1), nor by differences between the number of SNPs in genic and intergenic regions.

Estimates of additive and dominance genomic heritability from the separate analysis for each class of genomic regions are in Table 2 (Scenario 2). Results were similar to those obtained with additive kernels only (Fig. 1). These results corroborated that all genomic regions contributed to the marked additive and dominance genetic variation and, for BW and BM, synonymous regions had the greatest contribution to the additive genetic variance among all annotation classes. Relative to the estimates from the separate analysis in Fig. 1, for most genomic regions, there was a decrease in the estimates of the marked additive genomic heritability, which seemed to move to the marked dominance variance. Regardless of the trait under study, the contribution of dominance genomic variance to total genetic variation was negligible (~0.05) and the contributions of each class of genomic regions to dominance variance were almost the same.

**Table 2:** Estimates of additive and dominance genomic heritability of SNPs partitioned into six classes of genomic regions for body weight (BW), ultra-sound of breast muscle (BM) and hen house egg production (HHP)

Class of genomic regions	BW		BM		HHP	
	$h_{mA}^{2}$ ± SD	$h_{mD}^{2}$ ± SD	$h_{mA}^{2}$ ± SD	$h_{mD}^{2}$ ± SD	$h_{mA}^{2}$ ± SD	$h_{mD}^{2}$ ± SD
Intergenic	0.22 ± 0.04	0.05 ± 0.02	0.24 ± 0.05	0.07 ± 0.02	0.16 ± 0.04	0.05 ± 0.01
Intron	0.23 ± 0.03	0.05 ± 0.02	0.25 ± 0.05	0.06 ± 0.02	0.17 ± 0.04	0.06 ± 0.02
Missense	0.18 ± 0.03	0.04 ± 0.01	0.20 ± 0.03	0.04 ± 0.01	0.15 ± 0.03	0.05 ± 0.01
Synonymous	*0.28* ± *0.05*	0.04 ± 0.02	*0.28* ± *0.05*	0.05 ± 0.01	0.17 ± 0.04	0.05 ± 0.01
UTR	0.21 ± 0.04	0.05 ± 0.01	0.23 ± 0.04	0.07 ± 0.02	0.17 ± 0.04	0.06 ± 0.01
Up-down stream	0.22 ± 0.04	0.06 ± 0.02	0.26 ± 0.05	0.07 ± 0.02	0.18 ± 0.04	0.05 ± 0.01
All markers	0.23 ± 0.04	0.06 ± 0.02	0.26 ± 0.05	0.07 ± 0.02	0.17 ± 0.04	0.06 ± 0.02

Each genomic region was fitted separately

Numbers in italics indicate the largest estimates among all classes of genomic regions

$h_{mA}^{2}$ Additive genomic heritability, $h_{mD}^{2}$ dominance genomic heritability, *SD* posterior standard deviation

**Fig. 2:**
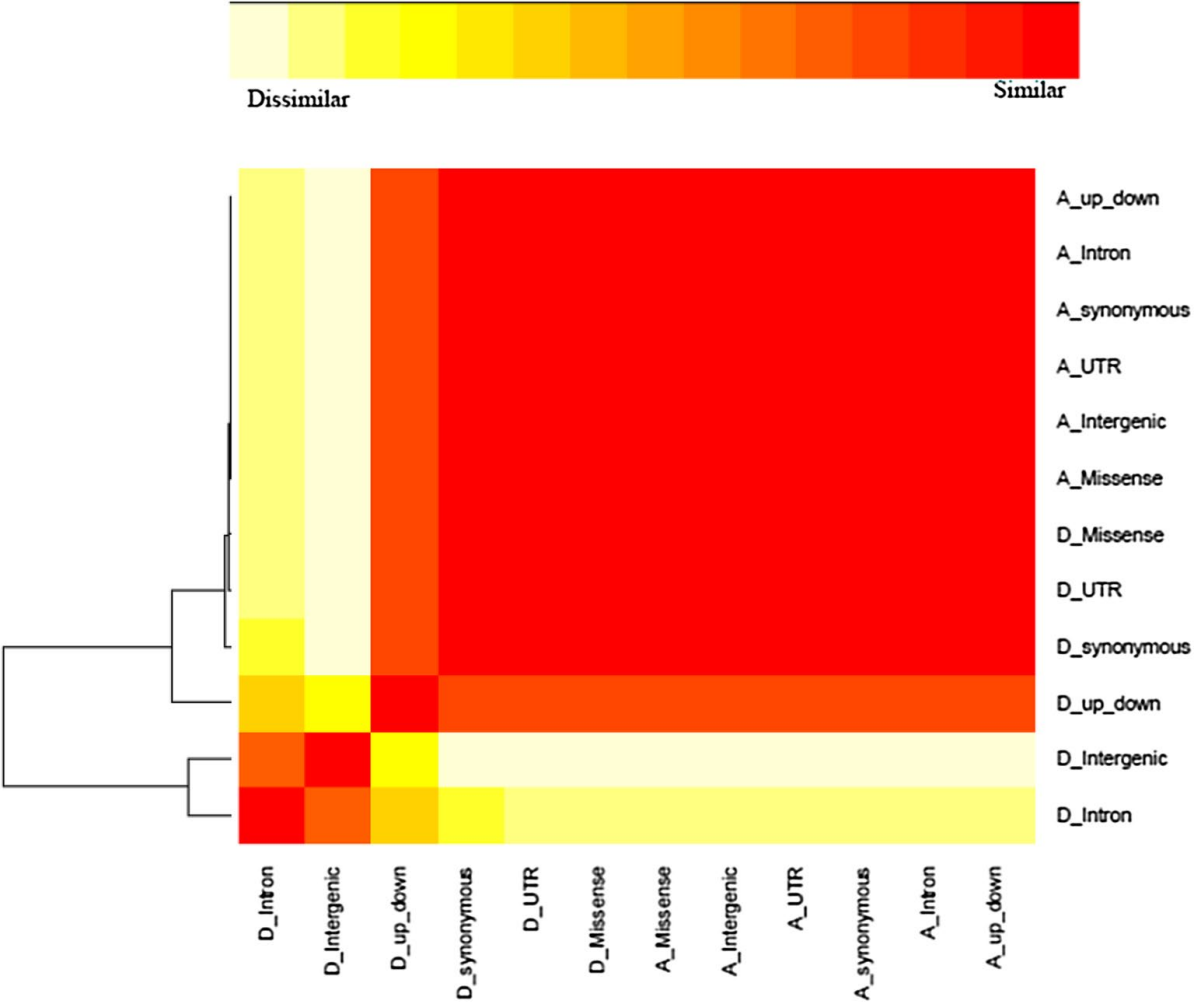
Heat map visualizing the degree of similarity between additive and dominance genomic relationship matrices for each genomic region. Very similar matrices are indicated in *red* and very dissimilar matrices are in *white*. Variable names that begin with an “A_” denote additive relationships and those with a “D_” denote dominance relationships. “*up*-*down*” indicates regions that are located 5 kb upstream and downstream of the gene

The heat map and a hierarchical clustering indicated high similarity between additive genomic relationships matrices that were computed by using SNPs within different genomic regions (Fig. 2). All genomic relationship matrices, except the dominance relationship matrices for intron and intergenic regions, were grouped in the same cluster. There was a large correlation between corresponding elements of **G** from different genomic regions (>0.90), with an average of 0.94. We also found that the correlation between corresponding elements of **D** was >0.83, with an average of 0.90. The correlation between additive and dominance relationships ranged from 0.70 to 0.82 with an average value of 0.76. Thus, these genomic relationship matrices are not orthogonal to each other, and there is some confounding between the estimated parameters. This clearly complicates the separation and interpretation of estimates. Genomic relationship kernels that are “orthogonal” to each other could probably enhance inference or prediction ability but such kernels are not straightforward to construct and further research is needed on this issue.

### Predictive ability

**Fig. 3:**
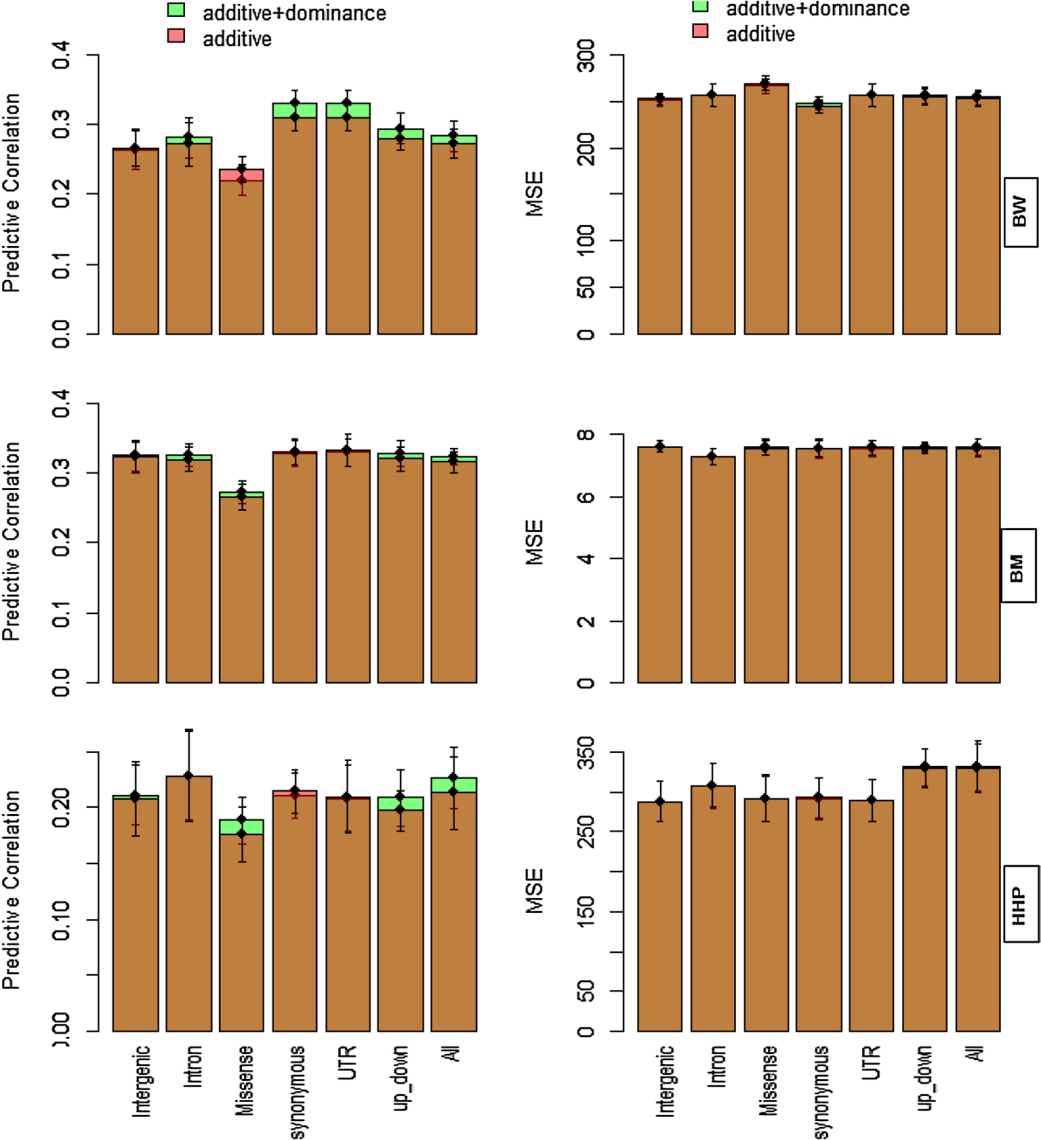
Predictive correlations and prediction mean-squared errors (MSE) resulting from different classes of genomic regions for body weight (BW), ultra-sound of breast muscle (BM) and hen house egg production (HHP) from the separate analysis of additive (**G**) and additive + dominance (**G** + **D**) kernels for each genomic region. The results were based on a fourfold cross-validation with 15 replications. “*up*-*down*” indicates regions that are located 5 kb upstream and downstream of the gene. “All” means that all SNPs were used to construct **G**. The *whiskers* represent 95 % confidence intervals and overlapping *bars* are in *bronze color*

Figure 3 represents the predictive correlation (left panel) and MSE (right panel) from the additive kernel (**G**) and additive and dominance kernels (**G** + **D**) for each genomic region [Scenarios (1), (2), respectively]. Here, each genomic region was analyzed separately, thus the LD between SNPs in different genomic regions can affect the results. The gain in predictive ability from the combined use of additive and dominance (**G** + **D**) kernels was negligible. In terms of predictive correlation, minor differences between classes of genomic regions were observed but, according to the MSE metric, all genomic regions performed similarly. For BW, when only additive or joint additive and dominance (**G** + **D**) kernels were fitted, the predictive correlation due to SNPs in synonymous and UTR genomic regions was greater than for other genic regions. The lowest predictive ability was obtained for the class of missense genomic regions. In general, for BW and BM, the predictive correlation obtained for different classes of genomic regions was similar. Overall, our results indicated that all classes of genomic regions influence the prediction of yet-to-be observed phenotypes.

For HHP, when additive and dominance kernels (**G** + **D**) were fitted jointly, the class of intron regions had the largest predictive correlation, but this superiority was not observed in terms of MSE. In agreement with genomic heritability estimates (Table 2), the MSE for genic regions was slightly smaller than for intron regions (HHP, Fig. 3).

**Fig. 4:**
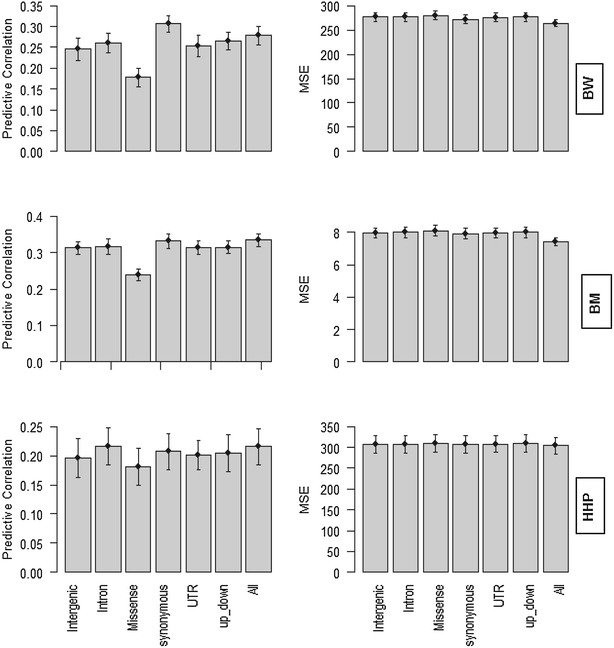
Predictive correlations and prediction mean-squared error (MSE) for the six classes of genomic regions for body weight (BW), ultra-sound of breast muscle (BM) and hen house egg production (HHP) from the joint analysis of all additive kernels $({\mathbf{G}}_{1} + {\mathbf{G}}_{2} + \cdots + {\mathbf{G}}_{6} ).$ The results were based on a fourfold cross-validation with 15 replications. “*up*-*down*” indicates regions that are located 5 kb upstream and downstream of the gene. “All” means that all SNPs were used to construct **G**. The *whiskers* represent 95 % confidence intervals

Predictive ability of the six classes of genomic regions for BW, BM and HHP when the statistical model fitted all genomic regions jointly is shown in Fig. 4. In agreement with the estimates of genomic heritability (Fig. 1), SNPs within the class of synonymous regions resulted in a better predictive correlation than other classes of genomic regions irrespective of trait, whereas in terms of MSE the predictive performance of the six classes of genomic regions was almost the same (Fig. 4). In terms of predictive correlation, the predictive abilities of the class of synonymous regions and of all sets of SNPs were the same whereas, in terms of MSE, using all SNPs resulted in a better predictive ability than when using any single class of genomic regions across all traits. The class of missense genomic regions led to the smallest predictive correlation.

## Discussion

Which parts of the genome contribute relatively more to the genetic variation of a complex trait is an important question in quantitative genetics. In human and dairy cattle studies, missense SNPs are over-represented in trait-associated variants e.g., [3], which is in agreement with their major role for protein sequence changes [34]. More recently, with the availability of transcriptomic data and findings from the ENCODE projects, it has been reported that 80 % of the genome has a biochemical function [1]. The statistical framework that was developed by Fisher [35] assumes that most traits are affected by an infinite number of genes and that each contribute very little to the variance of the trait, and are randomly distributed across the genome. Here, we partitioned the chicken genome into six classes of genic and non-genic regions and investigated their contribution to marked additive and dominance genetic variation, and to predictive performance. We found that all classes of genomic regions contributed to genetic variation and that this contribution was slightly greater for SNPs within synonymous regions. Variance component estimates can be regarded as measures of goodness of fit, but better fit will not necessarily lead to increased predictive accuracy for future samples because of issues such as model over-fitting. In terms of MSE, all annotation classes resulted in a similar predictive ability regardless of the trait under study. The MSE is a better and more flexible metric for comparing models than predictive correlations. The predictive correlations are bounded between 0 and 1, while MSE can move from 0 to infinity. Furthermore, MSE addresses both prediction bias and variability, whereas predictive correlations provide only a measure of association e.g., [36, 37].

Missense regions consistently yielded the lowest performance, whereas synonymous regions produced the highest performance in terms of predictive correlations. However, the predictive ability of genic regions with a few 1000 SNPs was more or less the same as that of non-genic regions with a hundred thousand SNPs. This agrees with the findings of Do et al. [11] who showed that predictive accuracy and prediction bias of genomic regions did not significantly differ from those of randomized SNP groups.

In agreement with the infinitesimal theory and with Morota et al. [12], we found that the whole-genome approach is a better choice for prediction than using genomic regions individually. In general, our results highlight the importance of having SNPs that cover the entire genome, which suggests that many nucleotides play a role in connecting genotypes to phenotypes.

Our results for BW and BM agree with the findings of Koufariotis et al. [9] who reported that synonymous parts of the genome explained a larger proportion of the additive variance than other genomic regions in dairy cattle. Importantly, recent studies have demonstrated some functional outcomes of synonymous mutations [10, 38]. In the fields of genetics and pharmacology, there is increasing interest for synonymous codon changes, which do not alter amino acids, [38, 39], since over 50 human diseases have been associated with synonymous mutations [39]. Furthermore, some studies have reported that synonymous codons can affect protein folding and function of translated proteins and, therefore, may be under selective pressure [40]. Other functions for synonymous mutations, such as the splicing of precursor mRNAs, alteration of the secondary structure of mRNA and effects on mRNA stability, have also been described [38, 39].

However, the association between SNPs in intron regions and complex traits cannot be ruled out since elements within introns have been demonstrated to have regulatory functions [41, 42]. Studies in human genetics have found that the DNaseI hypersensitivity sites are some of the most enriched regions for trait-associated SNPs [7]. Regulatory functions such as promoter, enhancer or transcription factor binding sites (TFBS) are located in the genomic regions of the upstream and downstream annotation classes, and Gusev et al. [7] reported that this part of the genome is responsible for part of the genetic variation in 11 human diseases. A slightly better predictive performance of the class of intergenic regions for broiler traits was also suggested in other studies e.g., [12], and hundreds of structural and copy number variants in intronic and intergenic regions were identified in the two Silkie and Taiwanese native chicken breeds [43]. In general, our results suggest that the variants within the non-coding genome are also important, thus attention should not be limited to variants within the protein-coding regions only.

Our heat map and cluster analysis showed a high level of similarity between genomic relationships based on SNPs in different genomic regions. Hence, if a study aims at partitioning the genetic variation into different classes of genomic regions or into additive and non-additive components, larger sample sizes must be used and non-orthogonality of genomic relationship matrices must be taken into account for proper interpretation. Increasing sample size may improve the power of detecting and distinguishing between minor additive and non-additive effects.

In agreement with our results, Yang et al. [8] partitioned the genome into genic and non-genic regions and found that genic regions explained more genetic variation than intergenic regions for height and body mass index (BMI) in humans. Morota et al. [12] found that, for BW and HHP, non-genic regions performed marginally better than genic regions, while for BM, genic regions resulted in a better predictive performance than non-genic regions. However, there are differences between our study and that of Morota et al. [12]. In the later, coding DNA sequences were clustered together and a tenfold CV design was used, while in our study, annotation classes were distinct and a fourfold CV layout was used. We also used a multiple-kernel approach, while in Morota et al. [12] a single-kernel approach was applied. A multiple-kernel approach helps to eliminate signals that one annotation class has on another annotation class.

We observed that additive genetic variance had a major contribution to total genetic variance. Several studies have reported that the contribution of non-additive variance to total variation is minor [44, 45], while evidence of interactions within and between loci is pervasive [45]. Mäki-Tanila and Hill [46] concluded that multilocus interactions make significant contributions to the additive variance and does not lead to large increases in the non-additive genetic variance, but if the heterozygosity level is high at multiple loci, epistatic loci explain a large part of the non-additive variance. However, Munoz et al. [14] showed that, for tree height, additive and non-additive components of the genetic variance were similar in magnitude. Quantifying non-additive genetic variance precisely requires the setting-up of orthogonal additive and dominance genomic relationship matrices (kernels) and the assumption of LE [47]. LD exacerbates non-orthogonality [18, 48] and construction of orthogonal kernels needs further investigation. Therefore, the decomposition of the variance obtained in this study via the two kernels (**G** + **D**) must be interpreted with caution, because we cannot rule out the possibility that a single kernel captures multiple sources of genetic variation. Morota et al. [18] reported that, for health traits in dairy cattle, the non-additive genetic variance contributed greatly to the genetic variance. However, our results showed that the contribution of dominance variance to total variance was negligible, and that all genic and non-genic regions represented similar contributions to the dominance variation captured by SNPs. Visscher et al. [49], using identity-by-descent (IBD) coefficients, showed that additive and dominance relationship coefficients were highly correlated (theoretical correlation was equal to 0.89), with an empirical correlation between additive and dominance relationships of 0.91. This illustrates that partitioning genetic variance into additive and dominance variance is indeed very difficult. In terms of predictive ability, our findings agree with a recent study on Fleckvieh cattle, where inclusion of dominance in the model did not increase the accuracy of predicted breeding (and total genetic) values of milk production and conformation traits [50].

There are some limitations in our study, i.e. (1) small sample size; although our sample of 1346 birds was sufficient to estimate the heritability for BW with a reasonable precision, for traits with a smaller heritability such as HHP (or for separating additive variance from dominance variance), larger sample sizes are necessary; (2) the precision of the annotated chicken genomic regions was maybe not sufficiently high, but one can assume that in the near future more precisely annotated data will be available; (3) a genomic annotation study using a ~300K SNP dataset cannot include all genic and non-genic SNPs; the availability of whole-genome sequence data is expected to identify all causal SNPs and, thus, results should be more accurate given the large size of the genotyped samples [51]. However, it has been argued that whole-genome sequencing data would not significantly improve predictive correlations using current genomic relationship-based methods (e.g., GBLUP or single step GBLUP) [52, 53]; (4) due to non-orthogonality between additive and dominance relationships, it was difficult to disentangle the additive variance from the dominance variance; with larger datasets, the issue of the correlation between additive and dominance coefficients should be less important, and additional non-additive effects, such as additive-by-additive or additive-by-dominance interactions could be estimated [49]; and (5) the variants that we investigated represent a set of SNPs that were selected to have a high MAF and to be evenly spaced across the genome (ascertainment bias), rather than a complete set of variants in the population. With the availability of whole-genome sequence data, this limitation should be alleviated.

## Conclusions

Our results provide information about the quantitative impact of coding and non-coding DNA regions on complex traits. All genomic regions regardless of the trait under study contribute similarly to the additive and dominance variances. However, the contribution of dominance variance to the total genetic variation was minor compared to that of the additive variance. In agreement with variance component estimates, the predictive ability of all genomic regions was similar except for the class of missense regions, which led to a lower predictive correlation. However, the whole-genome approach provided a better predictive ability than that obtained from classes of genomic regions considered individually.
